# Supplementary figures and images for: The chicken chorioallantoic membrane model for isolation of CRISPR/cas9-based HSV-1 mutant expressing tumor suppressor p53
Source: PLoS One. 2023 Oct 20;18(10):e0286231. doi: 10.1371/journal.pone.0286231 (PMC10588894; doi:10.1371/journal.pone.0286231)

# Schematic diagram of the shuttle vector construction for homologous recombination

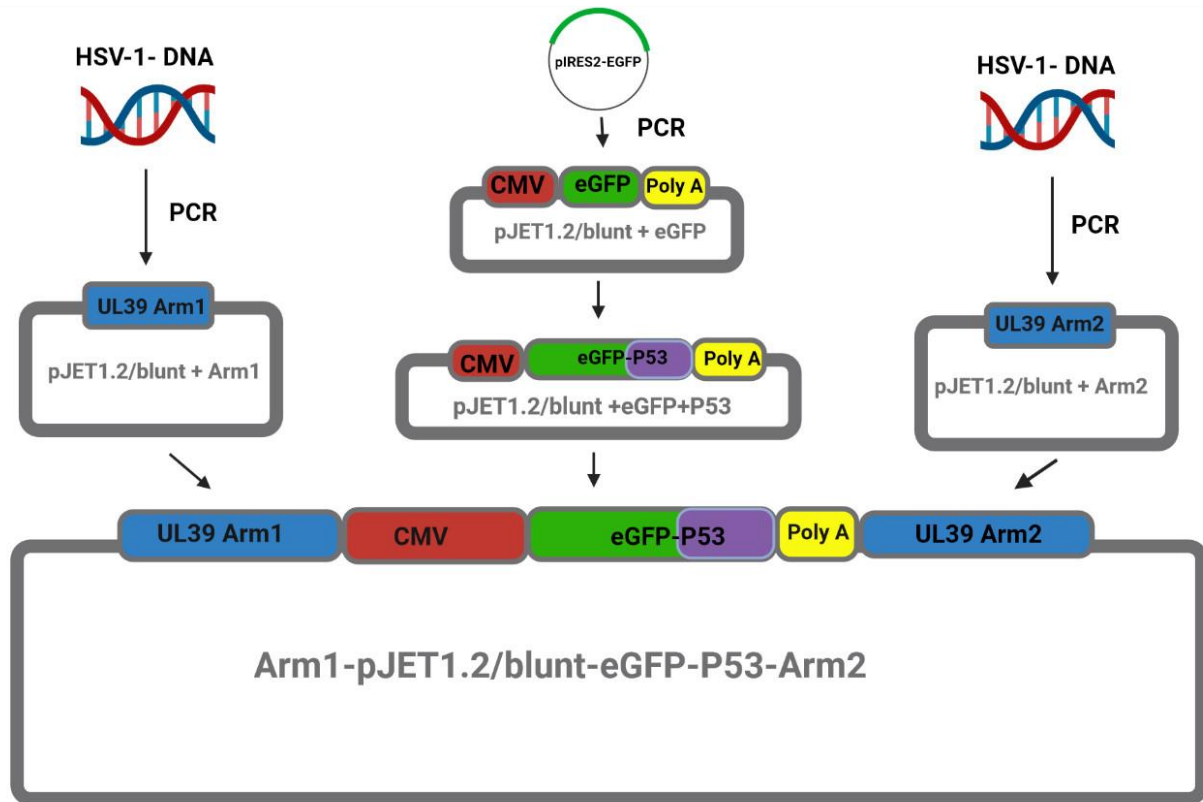

Supplement: S2 Fig — (PDF) [file pone.0286231.s002.pdf]
