# Supplementary material for: The chicken chorioallantoic membrane model for isolation of CRISPR/cas9-based HSV-1 mutant expressing tumor suppressor p53
Source: PLoS One. 2023 Oct 20;18(10):e0286231. doi: 10.1371/journal.pone.0286231 (PMC10588894; doi:10.1371/journal.pone.0286231)
Supplement: S1 Table — (PDF) [file pone.0286231.s003.pdf]

List of primers used in this study, related to the experimental procedures:

Primers used for sequencing of the UL39 gene of Δγ34.5/HSV-1 to design the gRNA

Gene	Sequence (5'-3') Forward	Sequence (5'-3') Reverse	GenBank No	Position
Upstream of UL39	TTCGTTGCGGTGACAAACAT	GCGCGTAGTTGAGAAGCCCAAA	JN555585.1	F: 86752-86771<br>R: 87577-87556
Downstream of UL39	GACTTTGGGCTTCTCAACTA	AGAGGGCGCTGAAGATATTG	JN555585.1	F: 87553-87572<br>R: 88513-88494

Primers used for cloning gRNA oligo's into Cas9 vector

Gene	Sequence (5'-3') Forward	Sequence (5'-3') Reverse	GenBank No	Position
SgRNA (UL39)	caccgTCTGGTGGTCGTAGAGGCGG	aaacCCGCCTCTACGACCACCAGAc	JN555585.1	F:88126-88107<br>R:88107-88126

Primers Used for generation of UL39 shuttle donor vector for homologous recombination (HR)

Target amplicon	Sequence (5'-3') Forward	Sequence (5'-3') Reverse	GenBank No	Position
Upstream homologous arm UL39	tatcagatctGGTGGTCCCTCAGCG	ccgaattcACTTGAACATTTCCCACCAC	JN555585.1	F: 87241-87255<br>R: 88096-88077
Downstream homologous arm UL39	tatcacgcgtgGCCATGCTGAACCTG	ctcctgcagTGTTCACCATCAGCAC	JN555585.1	F: 88144-88158<br>R: 88966-88951
CMV_P F- EGFP - Poly A R	ccgcgggaattcTAGTTATTAATAGTAA	gatatcacgcgtTAAGATACATTGATGAGTTT	plasmid<br>(Addgene, #6029-1)	F:1-16<br>R: 2217-2198

Primers used for recombination analysis

Target amplicon	Sequence (5'-3') Forward	Sequence (5'-3') Reverse	GenBank No	Position
**UL39 test**	**ACGACTTTGGGCTTCTCAAC**	**CCTTGTTTGTGGTGGCCTGG**	**JN555585.1**	**F: 87551-87570**<br>**R: 88222-88203**
