# Supplementary material for: The chicken chorioallantoic membrane model for isolation of CRISPR/cas9-based HSV-1 mutant expressing tumor suppressor p53
Source: PLoS One. 2023 Oct 20;18(10):e0286231. doi: 10.1371/journal.pone.0286231 (PMC10588894; doi:10.1371/journal.pone.0286231)

S1_raw_images for Figure 7. From left: lane 1: DNA ladder (100 bp), lane 2: ΔUL39/Δ34.5/HSV1-P53 recombinant virus (3592 bp), lane 3: Δ34.5/HSV-1 parental virus (671bp), lane 4: DNA ladder (1kb). The details are described in the legend of Figure 7.

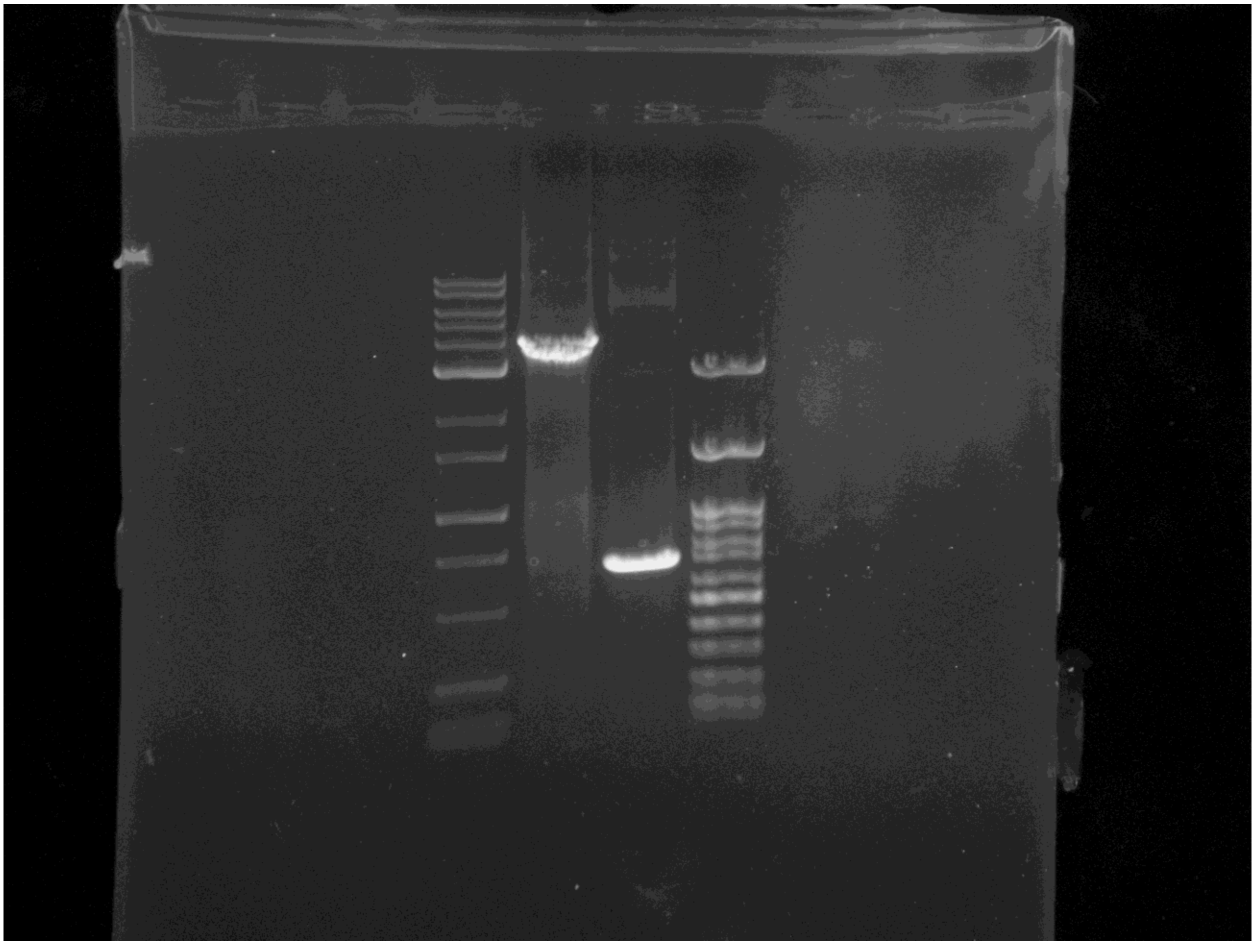

Supplement: S1 Raw images — (PDF) [file pone.0286231.s005.pdf]
