# Supplementary material for: The chicken chorioallantoic membrane model for isolation of CRISPR/cas9-based HSV-1 mutant expressing tumor suppressor p53
Source: PLoS One. 2023 Oct 20;18(10):e0286231. doi: 10.1371/journal.pone.0286231 (PMC10588894; doi:10.1371/journal.pone.0286231)
Supplement: Attachment — Submitted filename: Plos One Review.docx [file pone.0286231.s007.docx]

**The chicken chorioallantoic membrane model for isolation of CRISPR/cas9-based HSV-1 mutant expressing tumor suppressor p53**

The authors have made the suggested revisions and the comments were addressed in the response to comments. The revised manuscript shows improvements in the contents, details and organization.

Please see the minor specific comments on the revised manuscript

**Specific comments**

Line 51: Please clarify what is the conflicting data on apoptosis with the required reference.

Line 72: VSV: Expand “Vesicular Stomatitis Virus”

Line 126, 192, 218,233, 278: Replace the word “construction” with” rescue” or “generation”. Construction is usually used for describing vectors or plasmids.
